# Time-course gene profiling and networks in demethylated retinoblastoma cell line

**DOI:** 10.18632/oncotarget.4644

**Published:** 2015-06-25

**Authors:** Federico Malusa, Monia Taranta, Nazar Zaki, Caterina Cinti, Enrico Capobianco

**Affiliations:** ^1^ Laboratory of Integrative Systems Medicine (LISM), Institute of Clinical Physiology, CNR, Pisa, Italy; ^2^ Experimental Oncology Unit, Institute of Clinical Physiology, CNR, Siena, Italy; ^3^ College of Information Technology (CIT), United Arab Emirates University (UAEU), Al Ain, UAE; ^4^ Center for Computational Science (CCS), University of Miami, Miami, FL, USA

**Keywords:** retinoblastoma cell line, demethylation, gene expression profiling, co-expression networks, regulatory maps

## Abstract

Retinoblastoma, a very aggressive cancer of the developing retina, initiatiates by the biallelic loss of RB1 gene, and progresses very quickly following RB1 inactivation. While its genome is stable, multiple pathways are deregulated, also epigenetically. After reviewing the main findings in relation with recently validated markers, we propose an integrative bioinformatics approach to include in the previous group new markers obtained from the analysis of a single cell line subject to epigenetic treatment. In particular, differentially expressed genes are identified from time course microarray experiments on the WERI-RB1 cell line treated with 5-Aza-2′-deoxycytidine (decitabine; *DAC*). By inducing demethylation of CpG island in promoter genes that are involved in biological processes, for instance apoptosis, we performed the following main integrative analysis steps: i) Gene expression profiling at 48h, 72h and 96h after DAC treatment; ii) Time differential gene co-expression networks and iii) Context-driven marker association (transcriptional factor regulated protein networks, master regulatory paths). The observed DAC-driven temporal profiles and regulatory connectivity patterns are obtained by the application of computational tools, with support from curated literature. It is worth emphasizing the capacity of networks to reconcile multi-type evidences, thus generating testable hypotheses made available by systems scale predictive inference power. Despite our small experimental setting, we propose through such integrations valuable impacts of epigenetic treatment in terms of gene expression measurements, and then validate evidenced apoptotic effects.

## INTRODUCTION

Retinoblastoma is classified as “a paediatric ocular tumor that continues to reveal much about the genetic basis of cancer development” [1]. The genetic basis of the disease is originally centered on the biallelic inactivation of the *RB1* gene, which is relevant to all cases involving both the heritable form and most of the non-heritable cases. This mechanism confers limitless replicative potential to retinoblasts, thus implying that its loss leaves the cells without chromosomal stability. However, the same genomic instability does not seem to represent a hallmark in retinoblastoma as much as the epigenetic mechanisms do. The loss of expression of *RB2*, another member of the RB gene family, has been reported too, and it correlates with low apoptotic index and lesser differentiation in non-heritable cases [2].

A known player is *MYCN* through its amplification, and only for the non-heritable cases. *MYCN* encodes N-MYC, a transcription factor controlling the expression of cell cycle genes involved in promoting cell proliferation and regulating in particular the global chromatin structure through histone acetyltransferases (HAT), both in gene-rich regions and at sites far from any known gene [3]. By modifying the expression of its target genes, *MYC* activation drives apoptosis (down-regulation of the Bcl2 family), differentiation, and stem cell self-renewal. Interaction with other proteins in cancer paths have been illustrated by [4].

Among other identified oncogenes and tumor suppressors, some have become targets motivating the search for novel therapeutic solutions. Candidate driver oncogenes that were recently emphasized in retinoblastoma studies include the following genes: *MDM4, KIF14, DER* (chromatic remodeling factor), *E2F3,* a transcription factor and a tumor suppressor, *CDH11* (cadherin). These listed markers are collected in Table 1 of [1], with an assigned priority which is justified by the multiple evidences employed to validate them.

Relatively few studies have considered microarray-based differential expression in retinoblastoma, thus reporting evidence for up- or down-regulated gene sets. An indication which is coming from case - control studies refers to functional enrichment of several groups, in particular the DNA damage response pathway [5]. Due to the relevance of analyses inspired by pan-omics principles (see Figure 2 in [1]), an initial retinoblastoma map can be available, even if it appears not much integrated as in other cancers. We consider the importance of progressing in such key direction – integration - and confined our analysis to epigenetic features observed from targeted experimental evidences. Onco-epigenetic mechanisms are widely studied and believed to provide insight on cancer development and progression. Therefore, a very important role in therapy can be expected, by leveraging on the reversible nature of epigenetically-induced changes altering gene expression [6-8]. By causing changes in gene expression, epigenetic influences on phenotypes are possible, independently from changes in DNA sequence. DNA methylation is among such epigenetics-driven changes, and motivates the identification of markers regulating gene expression. When associated in promoter regions, gene silencing [9] is the observed mechanism, although other mechanisms are not completely known. Even if regarded as a chemical modification of cytosine bases associated with transcriptional repression, observational evidence at the transcriptional start sites (TSS) revealed only correlation with lack of transcription, remaining thus unclear whether it is a lack of activation or sign of repression which occurs [10].

Methylation analysis has been covered in retinoblastoma literature. An interesting contribution appears in the highlights of [11], looking at the role of the mutated *BCOR*. The incidence of *BCOR* mutation is relatively high, and since *BCOR* increases the methylation of H3K4 and H3K36, it influences the activation of transcription. It is known that DNA methylation represents a sort of gene-silencing mechanism for turning off genes and thus functionally re-organize genome data, in particular maintaining genome integrity and contributing to tissue-specific gene expression. Interesting genes were revealed as differentially methylated (see Table 5 in [1]), such as *RASSF1A* (tumor suppressor involved in microtubule stability), *MGMT, CDKN2* (tumor suppressor). The study in [12] reported a list of hypermethylated genes, in particular for *MSH6, CD44, PAX5, GATA5, TP53, VHL, GSTP1*. Also, in a recent study [13], the retinoblastoma genome was found to exhibit stability, suggesting that pathway dysregulation may be epigenetically-driven. Notably, the differential behavior of tumor and normal retinoblasts appears to a larger extent from the epigenetic rather than the genetic profile. Especially with *SYC* kinase, which is required for tumor cell survival, its inhibition brings the degradation of *MCL1* and caspase-mediated cell death, something observed both in cell cultures and *in vivo*. Also, WGS analysis revealed that 104 genes including 15 known cancer genes are differentially expressed, pointing in several cases to epigenetic deregulation. Finally, the non-coding genome has gained enormous attention in the discussion about the role of the altered epigenome features (see for instance [14]). Genome-wide studies in human cells have revealed the presence of long non-coding RNAs in amounts comparable to protein-coding genes. In particular, hundreds of sequence variants found in intergenic non-coding genomic regions have characterized a controversial field, the so-called “dark matter” [15-18], which might potentially be involved in regulation of gene transcription and epigenetic states.

Our study involves time course microarray experiments with the Weri-RB1 cell line treated with 5-Aza-2′-deoxycytidine (decitabine;*DAC*), a drug whose anti-tumorigenic effect regulates the epigenetic status of cells. Previously [19], the contribution of aberrant hypermethylation in retinoblastoma was demonstrated, suggesting that treatment with demethylating agents could represent a successful therapy. In particular, a tight correlation was observed between mutations located within a CpG-enriched region of the *RB2* gene, prone to de novo methylation, and its expression level in primary non-hereditable retinoblastoma. Methylation analysis of the gene from DAC treatment of the Weri-Rb1 cell line induced the re-expression of *RB2* and its related pro-apoptotic *E2F1*, *p73* and *p53* genes, thus highlighting a crucial role of epigenetic events.

This demethylating agent acts towards the correction of epigenetic defects, including reactivation of tumor suppressor genes silenced by epigenetic mechanisms in tumor tissues. By inducing demethylation of CpG islands in promoter genes that are involved in apoptosis and related biological processes, we analyzed the gene expression profiles at 48h, 72h and 96h after DAC treatment. In order to reconcile these evidences with those representing the state-of-the-art in retinoblastoma studies on markers, we designed a methodological approach centered on integrated bioinformatics tools. Aiming at integrability, evidence linkages only partially exist due to the heterogeneous multi-omic sources at play under different experimental conditions and various genomic scales. However, the evidenced gene profiles lead to further inference on functional enrichment and pathway annotations when integrated within regulatory contexts for our differentially expressed genes (DEG) and externally established markers, such as master regulatory (MR) gene paths and transcription factor (TF) driven protein networks.

## RESULTS

**Figure 1:**
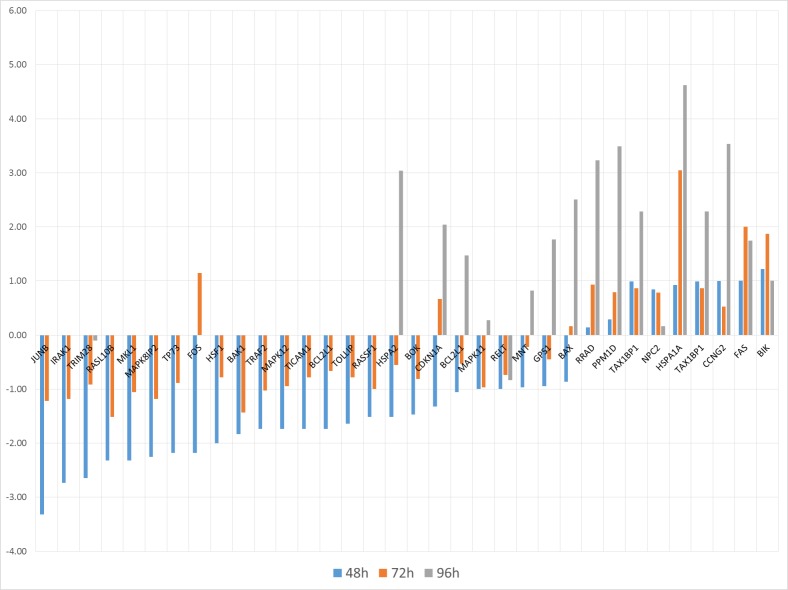

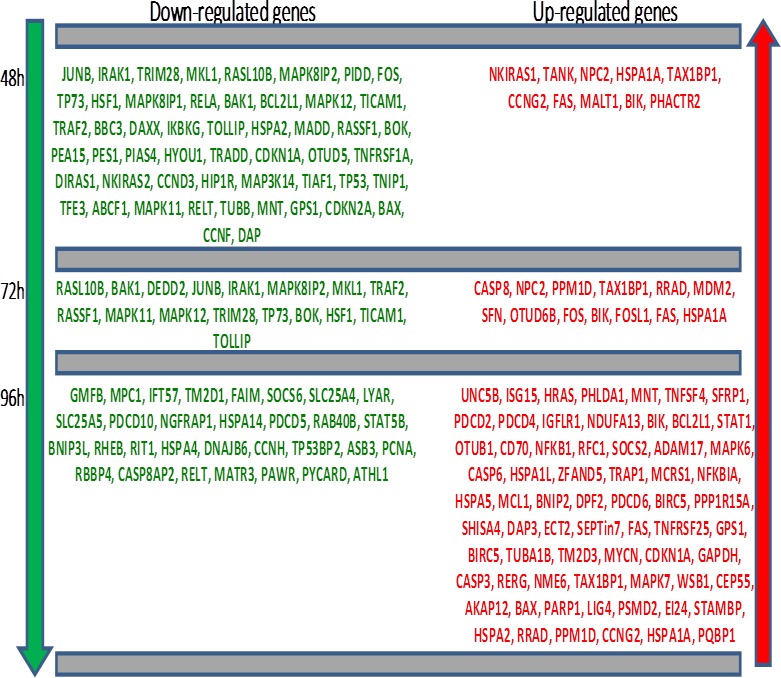
**A (top panel).** Barplot indicating gene-wide FC variation across times. **B (bottom panel).** Down-regulated and up-regulated DEG across times.

**Figure 2:**
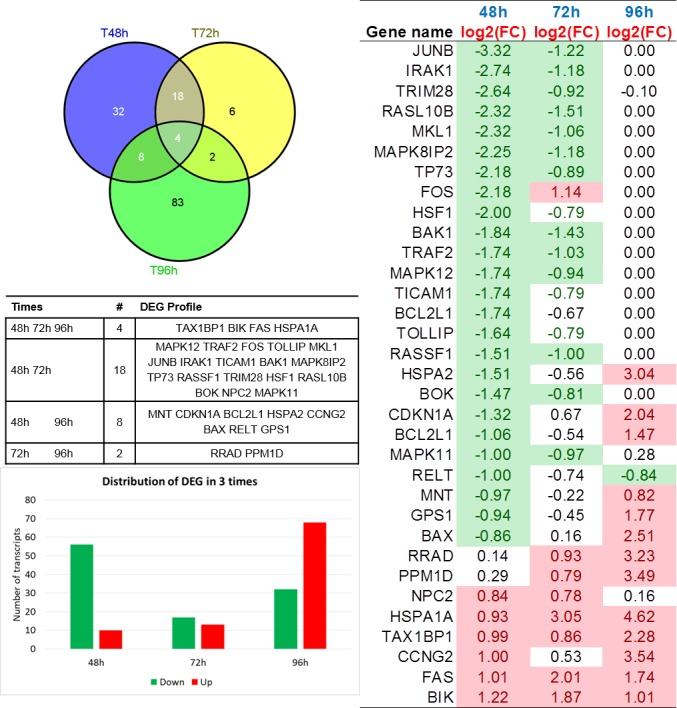
**Top-left: Venn Diagram of DEG in time course (48h, 72h, and 96h).** Mid-left table: DEG directionality in relation with intersecting genes; Bottom-left plot: barplot showing proportions of up- and down- regulated DEG across times; Right plot: log2(FC) DEG profiles heatmap. Only genes differentially expressed at least at 2 times appear.

DEG profiles measured at three times are presented in Figure 1A, with Fold Change (FC) fluctuating values. Some analytics are then reported in Figure 2, with a Venn diagram of time-specific versus time-overlapping DEG (see the embedded table below). Then, up-/down-regulated transcript amounts (bottom-left plot) are displayed, showing that proportions reverse within the time course. Individual temporal gene patterns (right plot) are listed shown, with an average taken in case of multiple probes for the same gene. The summary of the distribution of DEG across times is reported in Figure 1-B.

The main annotations in terms of GO biological processes (first panel) and pathways (second panel) are reported in Table 1 and Table 2, respectively. Enrichment is provided in corrected form too, and is reported for the most significant values, thus emphasizing the role of regulation of cell death and programmed cell death at each time point. Pathways show similarly that the top enrichment is always for apoptosis (including modulation and signaling), with other more or less specific entries such as NF-KB (48h), p53 (all times), TNF alpha (48h, 72h), Toll-like receptor (72h), plus other examples in part related to immune response.

**Table 1:** Annotated GO-BP The list is a selection from SM Table 1.

Term description	P-value	GENES	Bonferroni	Benjamini	FDR
regulation of cell death	4.31E-19	MNT, TUBB, BBC3, MADD, BOK, CDKN2A, TAX1BP1, TICAM1, PEA15, MALT1, DAP, HSPA1A, IRAK1, CDKN1A, IKBKG, TIAF1, MKL1, TRAF2, BAK1, TP53, BCL2L1, BAX, BIK, TRADD, RELA, FAS, DAXX, TP73, MAPK8IP1	1.42E-16	1.42E-16	5.84E-16
programmed cell death	5.37E-13	TIAF1, BBC3, PIDD, BOK, MADD, CDKN2A, TICAM1, TAX1BP1, PEA15, TRAF2, BAK1, TP53, INFRSF1A, DAP, BCL2L1, BAX, BIK, TRADD, FAS, TP73, DAXX	1.77E-10	8.83E-11	7.28E-10
regulation of cell death	1.44E-10	CASP8, MKL1, DEDD2, BOK, SFN, TICAM1, TAX1BP1, TRAF2, BAK1, HSPA1A, IRAK1, BIK, FAS, TP73, FOSL1	3.36E-08	3.36E-08	1.85E-07
programmed cell death	2.15E-07	CASP8, DEDD2, BIK, SFN, BOK, FAS, TICAM1, TAX1BP1, TP73, TRAF2, BAK1	5.02E-05	8.36E-06	2.77E-04
response to cytokine stimulus	2.02E-05	JUNB, CASP8, IRAK1, FOS, FOSL1	0.004696	5.88E-04	0.025949
regulation of cell death	9.75E-32	MNT, TM2D1, TNFRSF25, STAT1, SFRP1, LIG4, B24, PYCARD, TAX1BP1, BNIP31, NME6, DAP3, HSPA1A, STAT58, HSPA5, PAWR, CDKN1A, CASP8AP2, FAIM, BNIP2, CASP6, DPF2, NDUFA13, CD70, NFKBIA, PHLDA1, NFKB1, MCL1, PDCD6, SLC25A4, ADAM17, PDCD5, BIRC5, NGFRAP1, BCL2L1, TP53BP2, STAMBP, IFT57, BAX, BIK, FM, DNAJB6, CASP3, ECT2, SOCS2, HRAS	3.69E-29	3.69E-29	1.35E-28
programmed cell death	3.91E-28	TM2D1, TNFRSF25, STAT1, PDCD10, LIG4, PYCARD, MAPK7, TAX18P1, BNIP3L, NME6, DAP3, PAWR, CASP8AP2, FAIM, PDCD2, BNIP2, CASP6, DPF2, NDUFA13, NFKBIA, PHLDA1, NFKB1, MCL1, PDCD6, BIRC5, PDCD5, UNC58, NGFRAP1, BCL2L1, TPS36BP2, IFT57, BAX, BIK, PDCD4, FAS, CASP3, PPP1R15A, ECT2, HRAS	1.48E-25	7.39E-26	5.41E-25

**Table 2:** Annotated ClueGO pathways The list is a selection from SM Table 2.

GO Term (n. genes, source)	Term P-value corr. (Benjamini-Hochberg)	Associated Genes
	48h	
Apoptosis (14, WikiP)	−4.15E-18	[BAK1, BAX, BBC3, BCL2L1, BOK, CDKN2A, FAS, IKBKG, RELA, TNFRSF1A, TP53, TP73, TRADD, TRAF2]
NF-kappa B signaling pathway (12, KEGG)	2.30E-14	[BCL2L1, IKBKG, IRAK1, MALT1, MAP3K14, PIAS4, PIDD, RELA, TICAM1, TNFRSF1A, TRADD, TRAF2]
p53 signaling pathway (10, KEGG)	1.05E-12	[BAX, BBC3, CCND3, CCNG2, CDKN1A, CDKN2A, FAS, PIDD, TP53, TP73]
TNF alpha Signaling Pathway (9, WikiP)	3.21E-10	[BAX, BCL2L1, IKBKG, MADD, MAP3K14, TANK, TNFRSF1A, TRADD, TRAF2]
RIG-I-like receptor signaling pathway (8, KEGG)	1.30E-09	[IKBKG, MAPK11, MAPK12, OTUDS, RELA, TANK, TRADD, TRAF2]
	**72h**	
Apoptosis Modulation and Signaling (8, WikiP)	3.43E-10	[BAK1, BIK, BOK, CASP8, FAS, FOS, IRAK1, TOLLIP]
Toll-like receptor signaling pathway (7, KEGG)	3.32E-08	[CASP8, FOS, IRAK1, MAPK11, MAPK12, TICAM1, TOLLIP]
TNF signaling pathway (7, KEGG)	3.45E-08	[CASP8, FAS, FOS, JUNB, MAPK11, MAPK12, TRAF2]
p53 signaling pathway (6, KEGG)	5.36E-08	[CASP8, FAS, MDM2, PPM1D, SFN, TP73]
NOD1/2 Signaling Pathway (4, REACT)	3.57E-06	[CASP8, IRAK1, MAPK11, MAPK12]
Dimerization of procaspase-8 (3, REACT)	4.28E-06	[CASP8, FAS, TRAF2]
	**96h**	
Apoptosis Modulation and Signaling (11, WikiP)	2.70E-10	[BAX, BCL2L1, BIK, BIRCS, CASP3, CASP6, FAS, MCL1, NFKB1, NFKBIA, TNFRSF25]
Leptin signaling pathway (7, WikiP)	1.24E-06	[BAX, BCL2L1, HRAS, NFKB1, SOCS2, STAT1, STATSB]
p53 signaling pathway (7, KEGG)	2.27E-06	[BAX, CASP3, CCNG2, CDKN1A, E124, FAS, PPM1D]
Prolactin Signaling Pathway (7, WikiP)	4.29E-06	[CASP3, HRAS, NFKB1, NFKBIA, SOCS2, STAT1, STATSB]

### Apoptosis pathway landscape

Apoptosis represents an intracellular cell death program that counterbalances cellular proliferation, and maintains cellular homeostasis, while abnormal suppression affects cancer development and resistance to chemotherapy [20]. For instance, *BCL2* is an oncogene that suppresses cell death and antagonizes apoptosis by pro-survival proteins (so-called ‘guardians’) [21, 22]. The pro-apoptotic effectors *BAX* (*BCL2* associated) and *BAK* (*BCL2* antagonist) can be regulated independently from *BCL2* (say, from *TP53*), or by the BCL-2 family [21].

**Figure 3:**
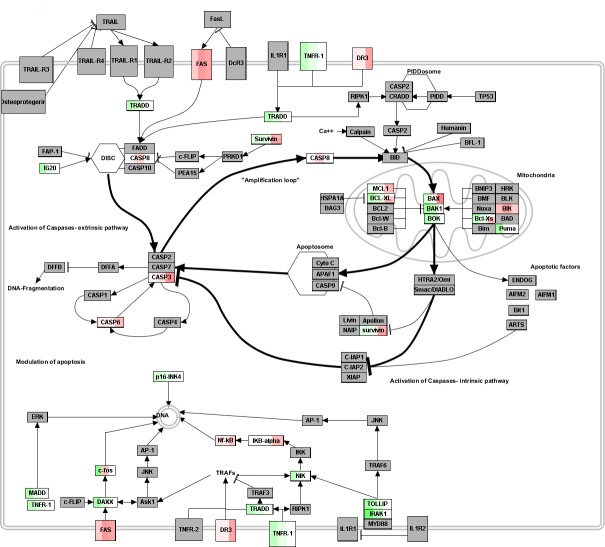
Apoptosis pathway landscape Red (up-) and green (down-regulated) DEG mapped from the different time profiles (gradient in the colored boxes is divided in 3 blocks, one per time point, from 48h to 96h). Annotation Source: Wikipathways; Graphical Tool: Pathvisio.

Interestingly, Figure 3 displays the apoptosis pathway landscape over which our DEG are mapped. Apoptosis induces multiple activations and regulations [23], and is mediated by intrinsic (triggered at intracellular level, say DNA damage, oncogene activation, etc.) and extrinsic (due to ligand binding at extracellular level) pathways. Clearly visible, the emphasis goes on the activation of caspase cascades. In particular, we evidenced that after DAC treatment the apoptotic signaling starts early with FAS and caspase 8 activation (48h and 72h after treatment, respectively), triggers caspase 3 and caspase6 extrinsic pathways, and also Bik and Bax mitochondrial intrinsic pathways (96h after DAC treatment), thus interfering with anti-apoptotic and pro-survival NF-kB and BCL2 intrinsic signals. These time-dependent patterns can be further elucidated graphically, by looking at the topology induced by such co-expressed genes. Since apoptosis evasion is considered a cancer hallmark, anticancer treatments mediate the cell death process through the apoptotic program whose interactome has a complex topological configuration.

### Apoptosis co-expressed sub-networks

**Figure 4:**
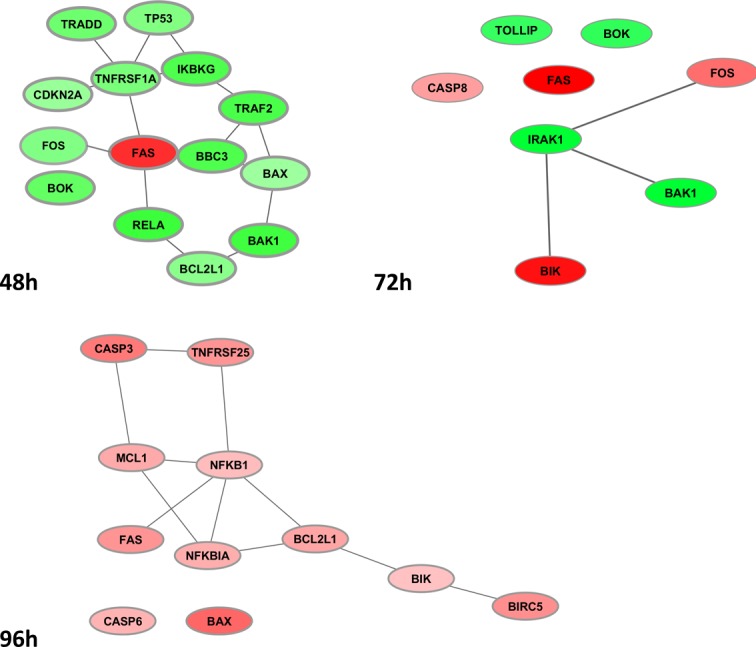
**Apoptosis co-expression sub-networks as from DEG profiles at 48h (top left), 72h (top right) and 96h (bottom).** Green/red nodes indicate down-/up-regulated DEG, respectively.

The sub-networks in Figure 4 report evidence snapshots of the time-dependent co-expression dynamics involved in apoptosis, with reference to only DEG sets. Connected as well as disconnected entities are displayed, and discussed below in detail. The interactome complexity results quite simplified by looking separately at each time. Thus, at 48h a densely connected down-regulated module is found, except from the up-regulated FAS, still centrally connected and known to play a central role in the physiological regulation of programmed cell death. At 72h, a small motif is observed, centered on down-regulated IRAK1 (Interleukin-1 receptor-associated kinase 1) and linked to the down-regulated pro-apoptotic BAK1, thus decelerating programmed cell death (it is known to bind to and antagonize the anti-apoptotic action of BCL2) and the up-regulated pro-apoptotic BIK (likely target of anti-apoptotic proteins, and whose function is to accelerate programmed cell death). Caspase-8 and FOS appear up-regulated too, again with FAS. Finally, at 96h, the up-regulated module is revealed, including FAS and the transcription factor NFKB-related genes, the latter being aberrantly activated in various cancers [24].

More in detail, the *FAS*-mediated module appearing at 48h is up-regulated and combined with i) Inhibition of anti-apoptotic activity exerted by *IKBKG, TRAF2, TRADD* ii) Inhibition of tumor suppressor activity exerted by *TP53* and *CDKN2A*, and iii) Inhibition of anti-apoptotic activity in *BCL2L1*, co-expressed with the connected *BAK1* and *BAX*. Interestingly, FOS (down-regulated) is implicated in cell transformation, but also associated with apoptotic cell death. At 72h, the down-regulated *IRAK1* appears as the only hub gene, the rest of them remaining singletons. Recruited to the IL-1 receptor complex by *TOLLIP*. *IRAK1* is a key mediator in the pathway IL-1R, initiating a cascade of signaling events which induce gene expression of inflammatory targets, and partially responsible for IL1-induced up-regulation of the transcription factor NFkB. Notably, *BAK1* and *BIK* are showing different regulation, suggesting variable acceleration of programmed cell death (i.e. only *BIK* increases its apoptosis-inducing activity), while also *FOS* is in active state of signal transduction, with likely effects on cell proliferation and differentiation.

At 96h, the two caspases responsible for apoptosis execution are activated, and the mediator of apoptosis *MCL1* connects to *CASP3*, likewise to *BCL2L1* via both *NFKB*1 and *NFKBIA*. Such dynamics involve *BIK*, which induces apoptosis by accelerating cell death, and *BIRC5*, member of the inhibitor of apoptosis (*IAP*) gene family, which encodes negative regulatory proteins that prevent apoptotic cell death. In particular, *MCL1* encodes an anti-apoptotic protein, which is a member of the BCL2 family, and mediates its effects by interactions with a number of other regulators of apoptosis. Since alternative splicing results in multiple transcript variants, *MCL1* is regulated in multiple ways involved in the regulation of apoptosis versus cell survival, and in the maintenance of viability but not of proliferation. Therefore, the interest in targeting its regulation is high, especially for development aspects, sustained growth and therapeutic resistance (observed in various cancers) [21].

Concerning the last sub-network, a special note is for *TNF* [25]. This is an important cytokine that plays a role in cancer, with two protein families implicated in the signaling mediated by the *TNF* receptor: 1) death-domain proteins, such as *TRADD, FADD, TNFR1, RIP*; and 2) TRAF domain-containing proteins, such as *TNFR2, CD40, TRAF1, TRAF6*. We only in part observed these patterns. *TRADD* recruits from the first group of proteins to activate signaling mediating *NFkB* and apoptosis. Other central signaling pathways are modeled by the interleukin receptor, for instance, and regulate *NFKB* activity (say, *TNFR1-TRADD-TRAF2-RIP*). Apoptosis is a critical function involved in the *TNF* pathway (say, *TRADD-RIP-FADD-TRAF2-CASP8*), and we can partially observe such signs (*TRADD-TRAF2* in the previous group, and *TRADD- TRAF2-CASP8* in the last group). In our experimental setting, the sequential activation of downstream caspases (at 96h) expected to be central to the execution of cell apoptosis, follows activation of *CASP8* (at 72h), which is involved in the programmed cell death induced by *FAS* and various apoptotic stimuli.

**Figure 5:**
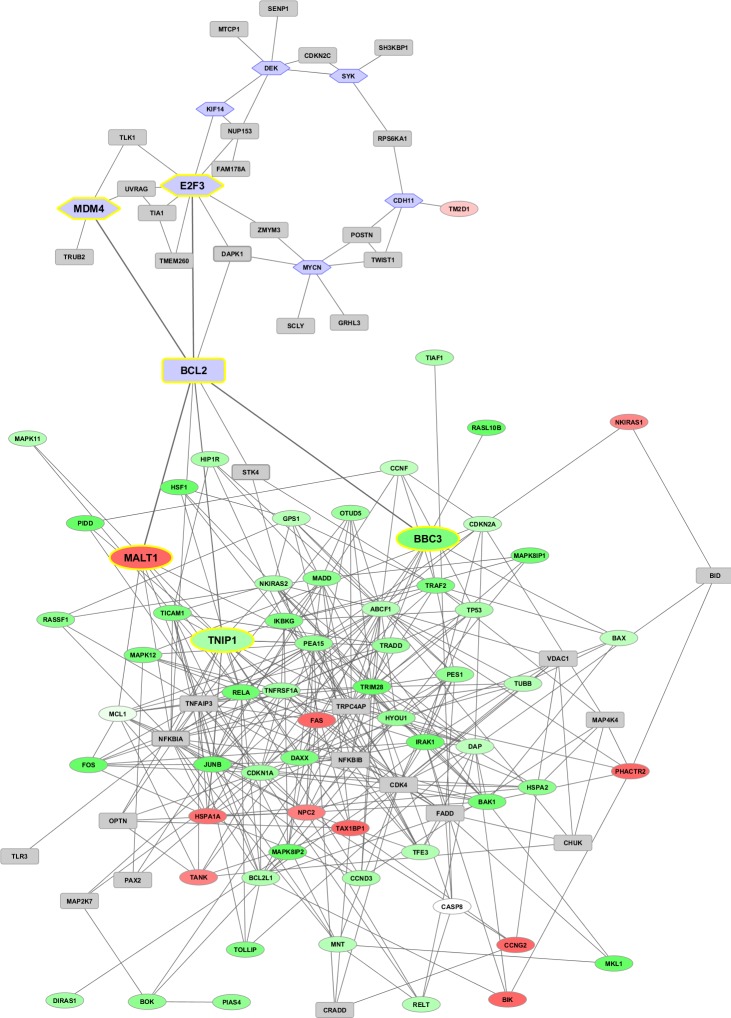

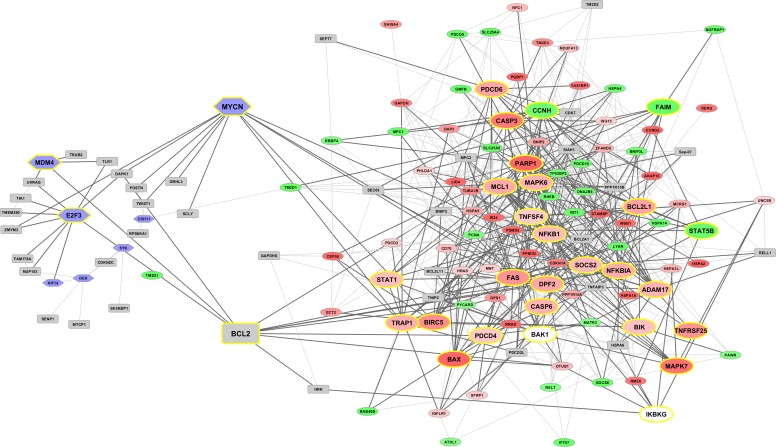
**Co-expression networks at time 48h (top) and 96h (bottom).** Networks from DEG (up-/down-regulated in red and green gradients, respectively) are interlinked with known marker genes (represented in hexagonal light blue frames) retrieved from the literature. Genes highlighted in grey squares with yellow border are directly linked to the bridge nodes, while generated interactors appear in grey squares.

**Figure 6:**
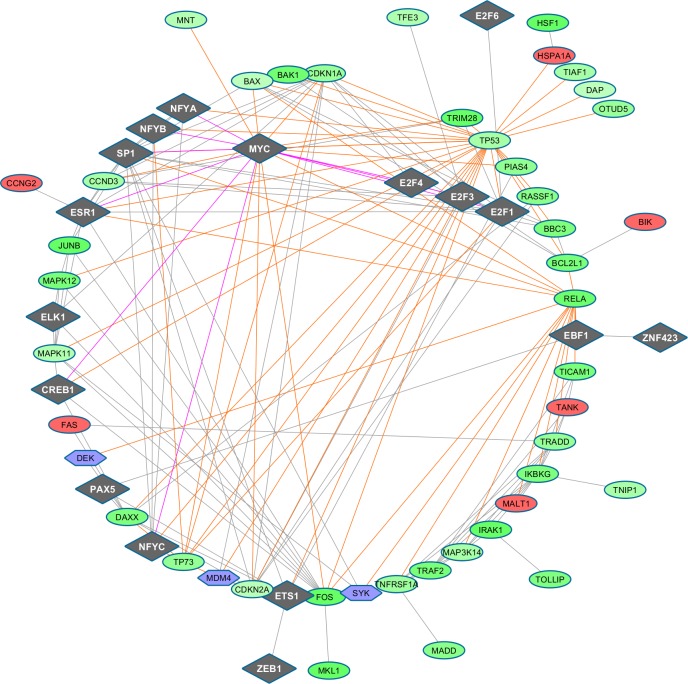

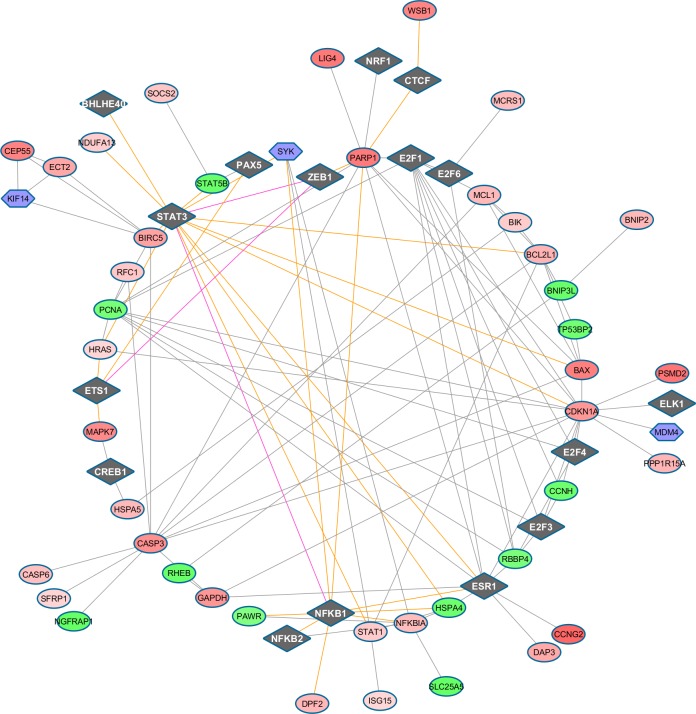

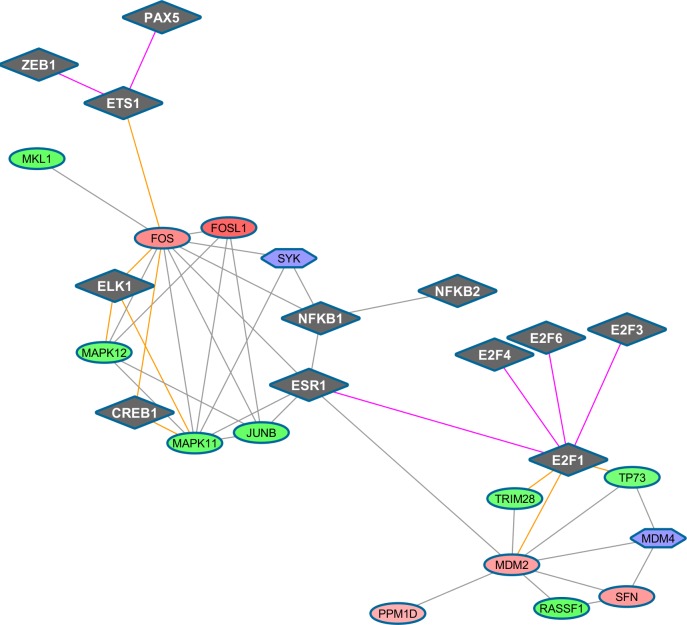
**TF-regulated PIN (confidence level 0.7) at time 48h (top panel), 72h (middle panel), 96h (bottom panel).** TFs appear as diamonds, while the other symbols remain as before.

**Figure 7:**
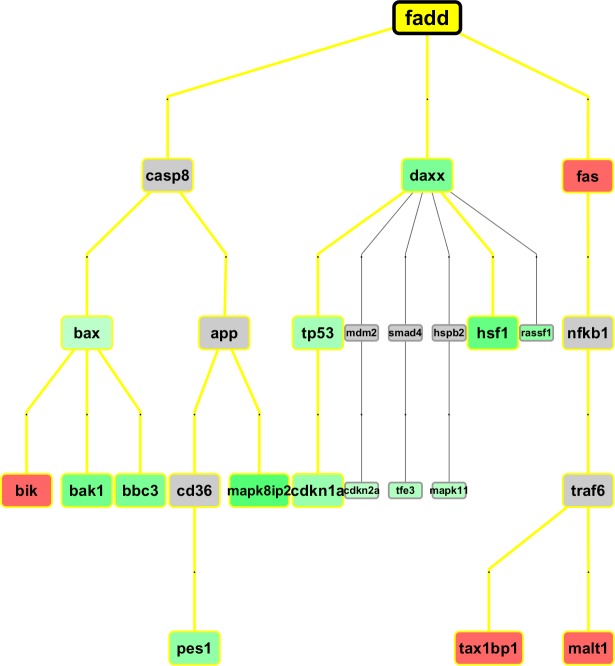

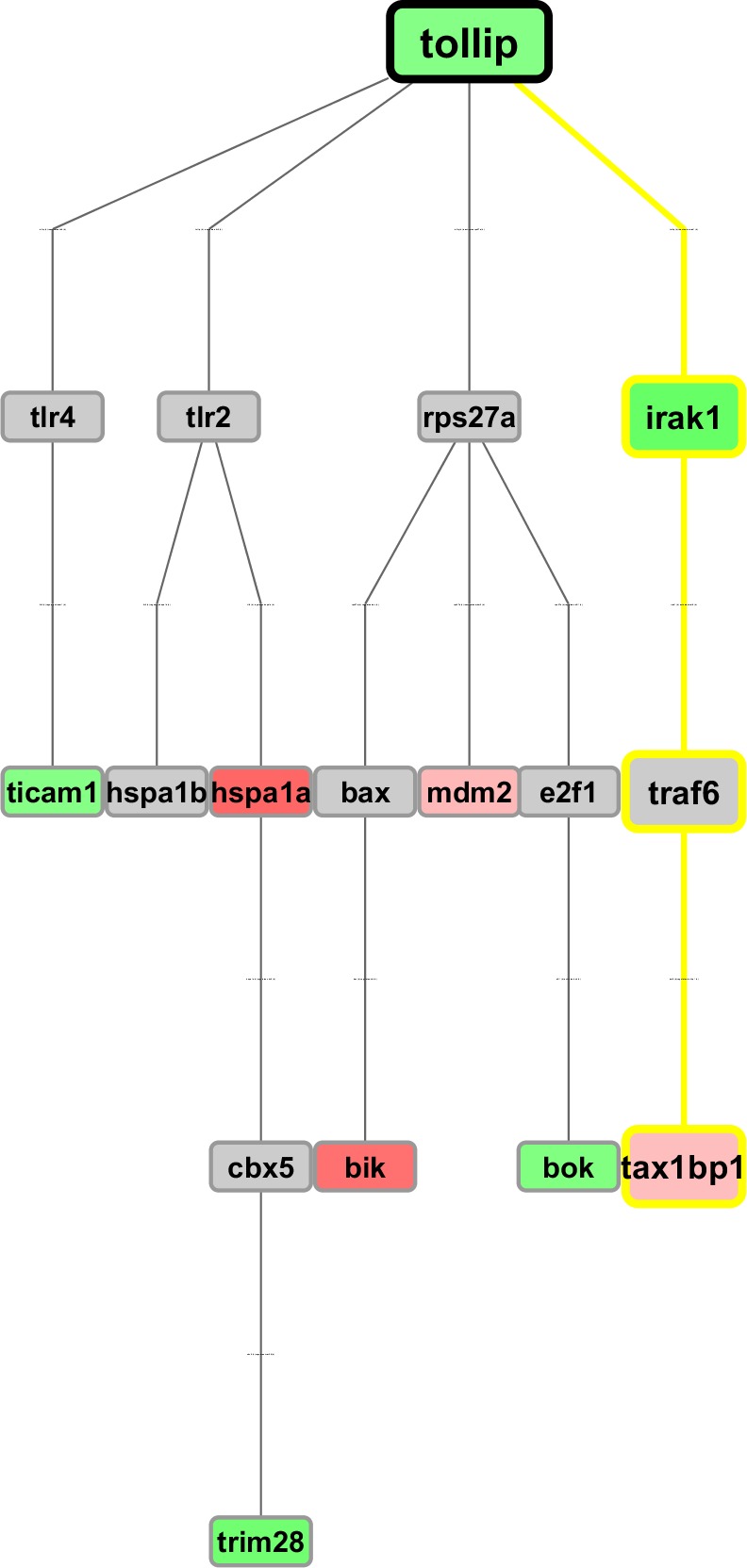

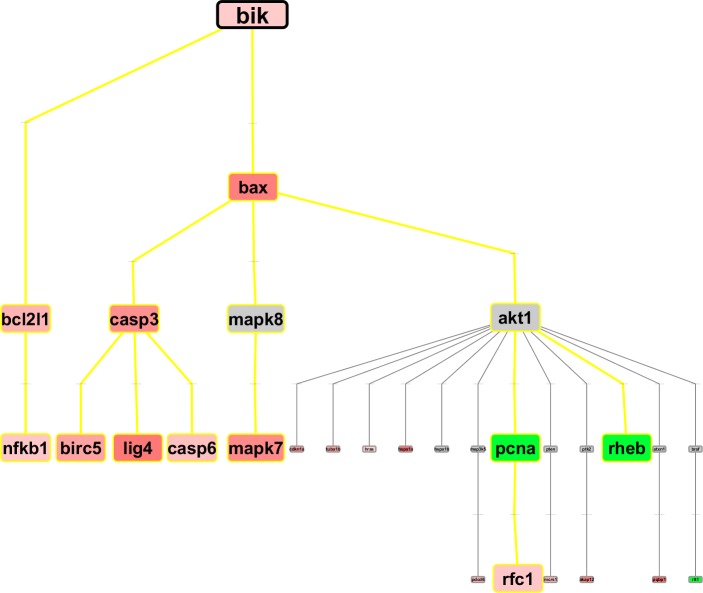
**MR hierarchical path examples at 48h (top), 72h (middle) and 96h (bottom);** these are the top-3 regulative graphs retrieved from curated knowledgebase in the GeneXplain web platform. Other examples have been made available in Supplementary Material.

Overall, a network can be broken down into modules, or groups of co-expressed genes, the function of which can be separated from that of other modules. The displayed sub-networks represent time varying network signatures of apoptosis which are reconstructed by only considering co-expression associations between our DEG. However, an interesting aspect is what they display, e.g. topological configurations that depend on treatment-induced dynamics. Therefore, these networks may be seen as potential fingerprints of particular disease phenotypes or treatment effects. More comprehensive co-expression maps will be illustrated in the next section through networks which are extended to all the computed DEG, and then integrated with other known markers (Figure 5), which are external to our experimental setting. Further differentiation of our regulatory maps will be finally provided, with the inclusion of transcription factors (Figure 6), and hierarchical paths (Figure 7). This way we aim to present a spectrum of network configurations elucidating relationships between evidences and supporting biological interpretation.

### Integrative marker analysis

The inference principle of integration is applied by first collecting evidences of marker genes which have been identified as candidate drivers of retinoblastoma progression [1]. In-depth analysis and review allow to build a reference knowledge base for linking our network-centered DAC-induced associations. The operation is repeated at all times to monitor possible changes in these associations in a co-expression context enriched also with interactors linking the two sets of markers genes (our DEG and externally evidenced).

### Co-expression networks

Two co-expressed network maps (Figure 5) involve DEG and reference marker/driver genes, together with predicted interactors inserted by the tool *GeneMania* [26] when direct links among nodes cannot be established. The top panel displays the connectivity map at 48h, in which the bridge node between the two sub-networks (one referred to DEG and the other to the reference markers) is *BCL2*. In particular, the *E2F3* and *MDM4* markers link to *BCL2*, which in turn links to *NFKBIA*, *MALT1* (strongly up-regulated), *BBC3* (strongly down-regulated), *TNIP1* (strongly down-regulated). At 72h, the network counts less DEG, and the marker *TM2D1* links the two sub-networks through both *CDH11* and *OTUD6B* (strongly up-regulated) link. This map can be found in SM Figure 1. At 96h, three bridge marker genes are found, *BCL2* (as in 48h), *TM2D1* (as in 72h), and the new entry *MYCN,* not directly linked to any marker, while some interconnected paths can be observed in the denser web region populated by highly expressed genes (*CASP3, PARP1, MCL1, MAPK6, BCL2L1, NFKB1, FAS*, et al.). Overall, the limitation of this type of analysis is well-known: co-expression interactions may or may not imply co-regulation dynamics. In order to consider regulation, we recover TFs and MRs associated to our DEG and markers, thus building protein-protein interaction networks (PIN) and hierarchical paths.

### Regulation maps

#### Tf-driven

Consideration of gene targets of TFs involves regulation activity that is quite complex to decipher, and also redundant. A common idea is to study the connectivity patterns between regulating and regulated entities, which in our case involve DEG. Assuming that the TFs may play important roles in retinoblastoma, and in the presence of epigenetic influences, the idea is to check what regulation patterns might be underlying our data. The potential of this analysis is huge, due to the fact that mammalian genomes encode about 1400 TFs [27]. However, the limitation is that the DNA sequence counterparts are known for just a fraction of them, and even less evidence is available for an assessment of the sensitivity to DNA methylation. Methylation can directly interfere with TF-DNA binding [9]. One possible reason is the presence of indirect repression mediated by methylated CpG binding proteins. In general, without this type of contextual evidence, it remains limited our understanding of regulation, including whether DNA methylation changes occur down or upstream of gene regulatory events [10]. We thus designed at each observational time point the same type of map represented as a PIN built from the tool *StringDB* [28], by selecting two confidence levels, and using the types of evidences available in the embedded knowledgebase to establish interactions between the proteins encoded by our DEG lists, the reference marker set, and with selected TFs.

Integrated TF and promoter analysis were performed using both the *Pscan* web tool [29] and the *GeneXplain* web platform (http://www.genexplain.com/), with the latter including a free version of *Transfac* [30] (release 2014.3;http://genexplain.com/transfac-1). In *Pscan* we analyzed our DEG list to detect over-represented TF binding site motifs at corresponding gene promoters, also considering all *Refseq* transcripts. *Jaspar* [31] profiles (http://jaspar.genereg.net) were used from a window bases width (−950,+50), with respect to the transcription start site (TSS). We analyzed 163 gene promoters covering 62 genes at 48h, 67 gene promoters covering 30 genes at 72h, and 210 gene promoters covering 97 genes at 96h. *Pscan* also uses a free *Transfac* version as descriptor, but we conveniently used *Transfac* from the *GeneXplain* web platform (public domain and more updated). Therefore, *Transfac* data were used from a workflow named “Identify enriched motifs in promoters (GTRD)” by using default input values and a promoter window bases width (−1000,+100), within TSS. We then considered a merged list of TF from both analyses (*Pscan/Jaspar* and *GeneXplain/Transfac*) and built an interaction network with *String-DB* at various confidence levels. The networks were exported in Cytoscape [32] for mapping the DEG list and the other markers. We illustrate below these scenarios, one for each of the 3 times at the highest confidence level 0.7, while the other maps computed at lower confidence level (0.4) are available from SM Figures 2-4. An interesting aspect is the coupling between DEG and known markers in these networks, offering the possibility to verify their joint regulation under the identified TF. We highlight examples of interesting paths of associations involving TF-*target proteins.*

#### Evidence at 48h

We discuss a couple of paths involving the bridge TF MYC, a multifunctional, nuclear phosphoprotein that plays a role in cell cycle progression, apoptosis and cellular transformation, and activates the transcription of growth-related genes, and the down-regulated DEG *RELA*, whose protein is involved in NFKB heterodimer formation, nuclear translocation and activation, known to modulate immune responses, and whose activation is positively associated with multiple cancers. *MYC* connects with down-regulated DEG *TP53* (tumor suppressor), *TP73* (participating in the apoptotic response to DNA damage), *FOS* (having an important role in signal transduction, cell proliferation and differentiation) and *CDKN2A* (inducing cell cycle arrest in G1-G2 phases, and acting as a tumor suppressor), among others.

#### Evidence at 72h

Very similar maps are obtained at the two confidence levels here considered. It is worth looking at the connectivity of the moderately up-regulated *FOS* regulating cell proliferation, differentiation, and transformation, and also associated with apoptotic cell death, with two TF groups: i) the group ELK1 (linked with the strongly down-regulated *IRAK1*), CREB1 (involved in different cellular processes), ETS1 (and then ZEB1 and PAX5, for which alterations in the expression of their genes are thought to contribute to neoplastic transformation); ii) the group E2F, via the moderately up-regulated *MDM2*, linked in turn to down-regulated (*TRIM28,* mediating transcriptional control by interaction with the Kruppel-associated box repression domain found in many TFs), *TP73* (participating in the apoptotic response to DNA damage), and *RASSF1* (potential tumor suppressor, required for death receptor-dependent apoptosis). *FOS* then links to three strongly down-regulated DEG, *MAPK11, MAPK12*, among the p38 MAPKs with an important role in the cascades of cellular responses evoked by extracellular stimuli such as pro-inflammatory cytokines or physical stress leading to direct activation of ELK1, and *JUNB*, involved in regulating gene activity following the primary growth factor response, together with the up-regulated *PPM1D*, member of the *PP2C* family of Ser/Thr protein phosphatases, negative regulators of cell stress response pathways.

#### Evidence at 96h

The association of the protein *PARP1* encoded by our strongly up-regulated DEG and involved in the base excision repair (BER) pathway, therefore in detection/signaling pathway leading to the reparation of DNA strand breaks, is with *CTCF* (chromatin binding factor involved in transcriptional regulation), a repressor, and STAT3, signal transducer and transcription activator mediating cellular responses to interleukin and growth factors. The latter link is established only via ZEB1, transcriptional repressor of interleukin 2 which connects with ETS1, this latter controlling the expression of cytokine and chemokine genes in many different cellular contexts, the differentiation, survival and proliferation of lymphoid cells, and regulating angiogenesis through regulation of expression of genes controlling endothelial cell migration and invasion. Other connections involve *BAX*, *CASP3*, NFKB1, pleiotropic and present in many cell types, and involved in signal transduction events related to inflammation, immunity, differentiation, cell growth, tumorigenesis and apoptosis.

The second example is leading to a multi-component path. The up-regulated DEG *CDKN1A* participates to *TP53* mediation, thus inhibiting cellular proliferation in response to DNA damage. Connectivity patterns then involve: a) ELK1, a nuclear target for the RAS-RAF-MAPK signaling cascade; b) *STAT1*, a moderately up-regulated DEG signal transducer and transcription activator mediating cellular responses to interferons (IFNs); c) STAT3; d) *BAX*, strongly up-regulated DEG antagonizing the apoptosis repressor *BCL2*, and promoting activation of *CASP3* (and thereby apoptosis); e) *PARP1*, strongly up-regulated and *CASP3*, moderately up-regulated and involved in the activation cascade of caspases responsible for apoptosis execution; f) *HRAS*, slightly up-regulated and functioning in signal transduction pathways by binding GTP and GDP; *g)* E2F1, belonging to the E2F family, controlling cell cycle and tumor suppressor proteins, and mediating cell proliferation and TP53-dependent apoptosis; h) *MDM4* (not a DEG, but an external marker) which inhibits TP53- and TP73-mediated cell cycle arrest and apoptosis by binding its transcriptional activation domain (also inhibiting degradation of MDM2); i) E2F4, controlling cell-cycle progression from G1 to S phase, and also possibly binding to RB1; l) *BCL2L1*, known potent inhibitor of cell death and inhibiting activation of caspases.

#### MR hierarchical paths

We used the *GeneXplain* web platform, and selected the workflow “Find master regulators in networks (*GeneWays*)” [33], an open integrated system combining molecular interactions from multiple sources of information and inferring a consensus network view. In particular, the tool tries to combine each gene into a hierarchical map through the mechanisms of regulation which are retrieved from a dataset of 479 distinct interaction modes, derived from a corpus of abstracts by using text mining and Natural Language Processing, and including a total of >6 ml interactions among 1,7 ml nodes (i.e. genes). We are looking at literature-curated regulations from mechanistic interactions. The workflow was adapted to our scopes by analyzing master regulatory molecules upstream of our DEG genes, with default input settings. The embedded gene network db is generated by computational text mining of >360,000 full text papers, and >8 ml publication abstracts, creating a hierarchical network of interactions among genes of interest and db ones. The hierarchical networks created in the master regulator analysis were exported as graphml files destined to Cytoscape for mapping the DEG values and for rebuilding the network with hierarchical layout, as in the initial tool, and with proper visualization of the selected DEG, and of the time-specific cascade relationships.

In the first of the three partial regulation paths (top plot), measured at 48h, we observe quite an abundance of detected DEG signals. At the top there is *FADD* - FAS-associated death domain – encoding an adaptor protein interacting with various cell surface receptors and mediating cell apoptotic signals. For instance, it binds to *FAS* (up-regulated), recruits and activates the initiator *Caspase 8,* which appears not expressed at 48h. Also proteins including *DAXX* (down-regulated) have been found to bind to *FAS* [19] (i.e. overexpression of *DAXX* enhances *FAS*-mediated apoptosis and activates the Jun N-terminal kinase (JNK) pathway). The *DAXX* apoptotic pathway is sensitive to both BCL2 and dominant-negative JNK pathway components and acts cooperatively with *FADD* [21]. Additionally, the disruption of the *MDM2–DAXX* interaction may be important for *p53* activation in response to DNA damage. *DAXX* represses p53 target promoters, and its co-expression with *MDM2* leads to further repression. DAXX can suppress cell death induced by *p53* overexpression and *p53*-dependent stress response, and is to be considered a negative regulator of p53 [21]). The suppressor p53 induces apoptosis or cell-cycle arrest in response to stresses.

In the second example, we report a master regulator path at 72h, centered on *TOLLIP*, a component of the signaling pathway of IL-1 and Toll-like receptors, and the encoded protein regulates inflammatory signaling. The closest DEG we have in an associated path is *IRAK-1*, encoding the interleukin-1 receptor-associated kinase 1. This gene is partially responsible for IL1-induced up-regulation of the TF *NFKB*. Notably, *Caspase 8* appears now expressed at 72h. Finally, in the third example we report *BIK*-centered regulation paths. Together with other death-promoting proteins, such as *BID, BAK, BAD* and *BAX*, *BIK* has pro-apoptotic activity, and interacts with anti-apoptotic members of the BCL2 family. Its activity is suppressed in the presence of survival-promoting proteins.

Finally, with reference to 96h, we find a good match with the evidences from the time-corresponding apoptosis sub-network, in which the interaction mode is different (i.e. co-expression). In the path of interest we can observe *BIC-BCL2L1-NFKB1* which is up-regulated, likewise the close path *BIK-BAX-CASP3-BIRC5*(or)*LIG4*(or)*CASP6*, which is again up-regulated.

### Validation

The signaling of apoptosis was considered for further validation, and both qRT-PCR and methylation-specific PCR (MSP) were employed to produce evidences. Given the multilevel regulation characterizing this death-inducing signaling complex, focus was on a few main entities, such as FAS, BIK, Caspase-8, whose mapping onto the MR hierarchical paths revealed significant modulation after treatment. For instance, Caspase-8 directly cleaves downstream caspases such as Caspase-3, or indirectly starts the activation of the intrinsic pathway of apoptosis. Caspase-8 represents a typical target for cancer therapy by acting though the Aza-induced restoration of its expression, and its silencing has been reported in neuroblastoma e medulloblastoma, for instance [34].

Epigenetic silencing is the designed strategy to induce Caspase-8 re-expression; through demethylation of the regulatory sequence of Caspase-8, the Aza agent induces transcriptional activation and sensitization to apoptosis [35-37]. Similar rationale for epigenetic silencing applies to our pro-apoptotic master regulators in order to induce their re-expression. In particular, validation with qRT-PCR for FAS, FADD, Tollip, Caspase 8, BIK and BAX has confirmed the signals obtained from microarray analysis (see evidences from SM qRT-PCR file) Also, the effect of DAC treatment on the patterns of promoter methylation has been further investigated for selected genes, i.e. FAS, Caspase-8 and BIK, thus providing further validation. As a result, methylation-specific PCR (MSP) indicates that FAS is not methylated in the Wery Rb1 cell line, thus leading to the conclusion that its up-regulation is not induced by treatment, but to indirect effects. Instead, Caspase-8 and BIK are methylated in the Wery Rb1 cell line, thus implying that up-regulation is due to a direct effect of the agent on the genes (SM MSP Wery cells file), and justifying transcriptional activation and sensitization to apoptosis of retinoblastoma cells.

## DISCUSSION

Our analyses refer to the assessment of temporal effects of DAC treatment on a retinoblastoma cell line. Weri-Rb1 is one of the few available cell lines (the other popular one being Y79) for this type of cancer, and remains important for the investigation of demethylation effects induced by treatment. Initially, we aimed to relate our findings to other evidences addressing genomic signals which were detected at different conditions and scales and thus containing distinct information. Networks are the best tool for this task. Since genomic signals appear correlated also on such scales [38], the associative dynamics between gene expression and DNA methylation can be influenced by scales, which in turn implies that it might not be possible to detect them under any particularly observable scale, maybe dependent on specific experiments, or we may lack measurements that allow to assess scale correlations. Under such general limitations, we measured demethylation effects at gene expression levels and built co-expression maps embedding various types of signals, from our DEG to other established markers, predicted interactors of the previous two entities, and then regulative entities too, such as TF and MR, all contributing to a map of connectivity patterns from which we aimed to analyze some interesting relationships.

Our time course analysis approach is relevant in two regards, at least: A) Profiles are available to assess whether the effect of epigenetic treatment is inducing particular effects not only through specific DEG sets, but also owing to some observed variation in expression levels in time which shapes co-expression network configurations; B) The integration of landmark entities obtained at different cellular states and experimental conditions, including epigenetic ones through our DEG, allows to measure wide-spectrum regulation effects, again in a network context. The role played by some genes is finally emphasized together with interlinked sub-networks, as these connectors or modulators of specific network activity involve regulation dynamics and build from observational evidence and predictions the integrative contexts for DEG and marker signals of special interest in retinoblastoma.

At a methodological level, integrative cancer inference from networks is becoming increasingly popular [39-46], and advances in computational and visualization tools [45] make it feasible (no need of a model) and effective (ability to establish relationships). In our case, the data diversity in terms of experimental sources and conditions are not preventing both maps and charts to be displayed, thus driving our inference approach. Since our generated evidenced are then validated to pinpoint the capacity of the proposed approach to produce valid testable hypotheses, we are confident that our application setting may turn out to be relevant for research in retinoblastoma, in particular owing to translational power linked to evidences at ensemble marker scale, which enables better elucidation of relevant aspects of epigenetic treatment with regard to complex multifaceted apoptotic signaling.

In conclusion, network analysis is an inference tool inherently able to describe associations that would not be predicted otherwise, to confirm through expected interactions relevant pathway cross-talks, and to identify unexpected correlative or causal relationships as a result of non-canonical gene interplay, due to disease. Biological systems contain complex relationships, including nonlinearities, which networks capture generally well, and since in such cases the presence of reference examples is crucial, the integration of evidenced markers from several experimental data sources and re-used data presents not only difficulties but also advantages, in particular when RNA expressions are complemented by methylation states information in order to decipher gene regulation.

## MATERIALS AND METHODS

### Culture cell line and 5-Aza-2′-deoxycytidine treatment

Retinoblastoma has been studied *in vitro* by the cell line Weri-RB1 treated with DAC (versus untreated one as control) in a time course experiment designed to analyze gene expression profiles at 3 specific times from treatment, i.e. after 48, 72 and 96 hours. Weri-Rb1 cells (ATCC, Rockinville, MD) were maintained in RPMI1640 medium supplemented with 10% foetal bovine serum and 2 mM L-glutamine, at split ratio of 1:2 once a weak. For treatments, cells were seeded at a density of 1 × 10^6^ cells/100-mm tissue culture dish. After 24 hours incubation, 2.5 μM 5-Aza-2′-deoxycytidine (Sigma-Aldrich) was added to the culture medium up to 96 hours.

### Cell viability and FACS analysis

Quantitative cell viability was measured by colorimetric assay using a cell proliferation kit (MTT) (Roche Molecular Biochemicals). Treated and untreated Weri-Rb1 cells were grown in microtiter plates (96-well) in a final volume of 100 μl culture medium. Cells were incubated for 48, 72 and 96 hours in the presence or absence of the 2.5 μM 5-Aza-2′-deoxycytidine. Cell viability was expressed as the percentage of the absorbance of drug-treated and untreated cells relative to that of the untreated at 0 hours. FACS analysis (Becton–Dickinson FACScan) was carried out on treated cells and compared to those untreated after 48, 72 and 96 hours of culture.

### RNA preparation

Total RNA samples were isolated from treated and untreated Weri-Rb1 cells after 48, 72 and 96h of cell cultures using TRIZOL reagent (Invitrogen, CA, USA) according to the manufacturer's instructions. Concentration of purified RNA samples were determined by A260 measurement and the quality was checked by Lab-on-a-chip analysis (total RNA nanobiosizing assay, Agilent) with the Agilent 2100 Bioanalyzer.

### cDNA microarray experiments

RNAs isolated from treated and untreated Weri-Rb1, and transcribed in cDNAs, were used to carry out the microarray analysis using a microarray chip from Miltenyi Biotech named PIQOR^TM^ Cell Death Human Sense Microarrays which contain 200-mer oligo-probes covering 494 human genes. In some cases, multiple probes are present, possibly allowing isoform detection. Interpretation of gene expression microarrays requires a mapping from probe set to gene. A given gene may be detected by multiple probe sets, thus leading to some inconsistency of measurement. When microarrays are used to identify DEG associated with a biological phenotype, a probe set with an expression pattern of interest can be mapped to a particular gene or set of transcripts. A usual way to estimate a single expression value for a particular gene is through the average expression value of all probe sets that map to the gene. Spots flagged as low quality were excluded from further analysis. Hybridisation, scanning and data analysis were performed as described in detail (1). Briefly, image capture of hybridised PIQOR^TM^ microarrays were done with the laser scanner ScanArrayTM Lite (PerkinElmer Life Sciences); mean signal and mean local background intensities were obtained for each spot of the microarray images using the ImaGene^TM^ software (Biodiscovery).

### Microarray analyses

Detection of the expression levels of transcripts in the 3 time profiles was achieved by using a Cy5/Cy3 custom platform designed PIQOR from Miltenyi Biotech and containing almost 500 genes related to apoptosis, cell death and inflammation. Local background was subtracted from the signal to obtain the net signal intensity and the ratio of Cy5/Cy3 was calculated. Subsequently, the mean of the ratios of the four corresponding spots representing the same cDNA was computed. The ratios were normalized using the Median and the Lowess methods. As an additional quality filtering step, only spots/genes were taken into account for the calculation of the Cy5/Cy3 ratio that have at least in one channel a signal intensity that was at least 2-fold higher than the mean background. We considered the selection of down-regulated based on genes with an expression ratio below 0.58, while up-regulated genes have values over 1.70. The microarray chip from Miltenyi contained 4 technical replicates and a quality control implemented in the analysis taking into account the coefficient of variation (CV = σ/μ) as a parameter referring to the quality of replicated spots, expressed as a percentage and complementing the information from expression ratios (see SM Table 3 for details).

### DEG, pathways and networks

From the DEG profiles analyzed as individual gene sets, we considered as an important aspect their connected representations. *GeneMania* was used to generate the networks, showing co-expression dynamics among the connected genes. When launching the web application, the input settings in default mode were selected from *GeneMania* to build the interactomes. In the *Networks* section, the available Co-expression, Co-localization, Genetic interactions, Pathway, Physical interactions, Predicted and Shared protein domains were used with default setting. In the *Network Weighting* section the automatically selected weighting method was used. In the *Number of gene results,* choice was for display of 20 related genes and at most 10 related attributes. These results were imported into *Cytoscape* to build the maps for display. *GeneMania* allowed to assess interactions occurring at co-expression level for the DEG sets in all the measured time course profiles, and the network configurations were built from the log expression ratio values.

Functional enrichment analysis was obtained by the *David* [47] (http://david.abcc.ncifcrf.gov/) and *PantherDB* (http://www.pantherdb.org/) [48] tools for GO enrichment and pathway mapping, respectively. Pathway analysis was performed using *ClueGo* (http://apps.cytoscape.org/apps/cluego) [49] in Cytoscape 3.1, and by selecting standard tools for annotation such as Wikipathways (http://www.wikipathways.org/index.php/WikiPathways) [50], KEGG (http://www.genome.jp/kegg/) [51], and Reactome (http://www.reactome.org/) [52] as pathway knowledge basis. In particular, the Apoptosis pathway map was downloaded from Wikipathways (http://www.wikipathways.org/index.php/WikiPathways) and then imported in *PathVisio* (http://www.pathvisio.org/) [53] in which the expression ratios corresponding temporal gene profiles were mapped. The *Clue*GO settings for pathway analysis included 3 genes/min and 4% genes in the advanced term option, with kappa score set at 0.4. The statistical analysis settings included right sided hypergeometric test for the enrichment with Benjaimini-Hochberg *p*-value correction. Integrated transcription factor and promoter analysis were performed using both the *Pscan* web tool (among other explored, such as *Opossum* (http://opossum.cisreg.ca/oPOSSUM3/), etc. [54]) and the *GeneXplain* platform. The transcription factor networks were built using *String-DB* at various confidence levels (the highest set at 0.7). The networks were exported on Cytoscape for mapping the DEG. Master regulator networks were built inside *GeneXplain* web platform by using *TransPath* knowledge base (http://genexplain.com/transpath-1) [55]. The *Geneways* knowledge base available from the tool was selected with default values for input analysis. The hierarchical networks created in the master regulator analysis were exported as graphml files in order to be handled within Cytoscape for mapping DEG values and rebuilding the network with hierarchical layout, as in the main analysis, and with proper visualization highlighting selected DEG and cascade relationships along all the profiles.

Other related information is available from SM Tables 4-5 (interactions in co-expression networks), SM Tables 6-7 (selection of TFs), and SM MR folder (top 3 regulation paths selected at each time).

### Quantitative RT-PCR experiments

qRT-PCR was performed to quantify mRNA levels in some of the relationships evidenced in particular by regulatory paths governed by master regulators. Total RNA was extracted from Weri-Rb1 cells using NucleoSpin RNA isolation kit (Macherey-Nagel) according to the manufacturer's instructions. RNA concentration and purity was determined by Picodrop spectrophotometer. For each sample, 1 mg (micro) of total RNA was reversely transcribed using the Maxima H Minus First Strand cDNA Synthesis Kit (Thermo Scientific). Gene expression was determined by DyNAmo Flash SYBR Green qPCR Kit (Thermo Scientific), using the PikoReal Real-Time PCR System (Thermo Scientific). All samples were analysed in triplicate.

#### Primers used

*b-ACTIN*: sense 5′-TGCGTGACATTAAGGAGAAG-3′, reverse 5′-GCTCGTAGCTCTTCTCCA-3′;

*FAS:* sense 5′-AAAGCTAGGGACTGCACAGTCA-3′, reverse 5′-GTCCGGGTGCAGTTTATTTCCA-3′;

*BAX:* sense 5′-TTTTCCGAGTGGCAGCTGACAT-3′, reverse 5′- TTCTGATCAGTTCCGGCACCTT-3′;

*BIK:* sense 5′-TGGAGGTTCTTGGCATGACTGA-3′, reverse 5′-ACTGCCCTCCATGCATTCCAAA-3′;

*TOLLIP*: sense 5′-TGGCCAAGAATTACGGCATGAC-3′, reverse 5′- ACCGTGCAGTGGATGACCTTAT-3′;

*FADD*: sense 5′-CAGAGAAGGAGAACGCAACAGT-3′, reverse 5′-AGGTAGATGCGTCTGAGTTCCA-3′;

*CASP8*: sense 5′-TTTCTGCCTACAGGGTCATGC-3′, reverse 5′-TGTCCAACTTTCCTTCTCCCA-3′,

#### One-way ANOVA

Amplification conditions were: 7 minutes at 95°C followed by 40 cycles of 10 seconds at 95°C, 20 seconds at 60°C and 20 seconds at 72°C. The experiments were performed in triplicate. The relative expression of target genes was evaluated using the comparative cycle threshold method, with b-actin used for normalization. For the significance of gene expression values, the statistical analysis of ΔCt values was based on ANOVA (*p* < 0.05; mean ± sem), reported in SM qRT-PCR file.

### Methylation-specific PCR (MSP)

DNA methylation patterns in the CpG islands of FAS, CASP8 and BIK were assessed by MSP, on the basis of the sequence differences between methylated and un-methylated DNA after sodium bisulfite modification. Genomic DNA was extracted from Weri-Rb1 cells and subjected to bisulfite modification by the Thermo Scientific EpiJET Bisulfite Conversion Kit. Successively, the modified DNA was used for MSP reactions. The primer pairs specific for methylated (M) and un-methylated (U) sequences were the following:

FAS:

M sense 5′-AGTTTCGGCGTTTTTCGGAGATTATTGC-3′

M antisense 5′-CACCCGCGCCGAAACGAACC-3′

U sense 5′-GGTAGTTTTGGTGTTTTTTGGAGATTATTGT-3′

U antisense 5′-CACCCACACCAAAACAAACCTTTAAC-3′

CASP8:

M sense: 5′-GTT GGT TTT ATT TAG TTC GGC-3′

M antisense: 5′-CCC TAT CGA TAA CAA ATA ATA TAC-3′

U sense: 5′-GTT GGT TTT ATT TAG TTT GGT-3′

U antisense: 5′-CCC TAT CAA TAA CAA ATA ATA TAC-3′

BIK:

M sense 5′-GGGAGTCGTGTTTAGGTTTTATC-3′

M antisense 5′-GAACAAAAAAAATACGTTTCGAA-3′

U sense 5′-GGGGAGTTGTGTTT AGGTTTTATT-3′

U antisense 5′-CAAACAAAAAAAATACATTTCAAA-3′

PCR products were separated on a 2,2% agarose gel containing ethidium bromide and visualized under ultraviolet illumination.

## SUPPLEMENTARY MATERIAL FIGURES AND TABLES




















