# Supplementary material for: Time-course gene profiling and networks in demethylated retinoblastoma cell line
Source: Oncotarget. 2015 Jun 25;6(27):23688–707. doi: 10.18632/oncotarget.4644 (PMC4695145; doi:10.18632/oncotarget.4644)
Supplement: Supplementary file 1 [file oncotarget-06-23688-s001.pdf]

Time-course gene profiling and networks in demethylated retinoblastoma cell line

Supplementary Material MR

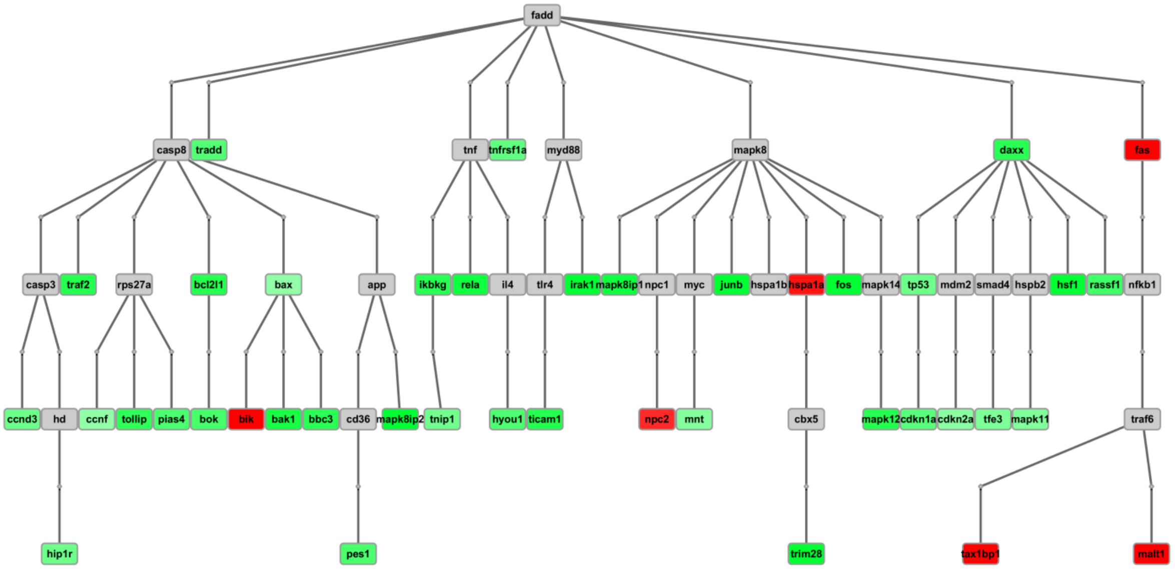

48h FADD with DEG

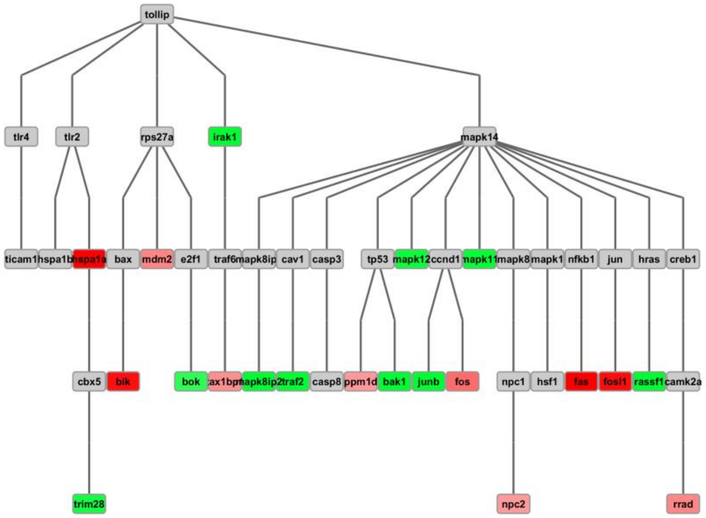

72h TOLLIP with DEG

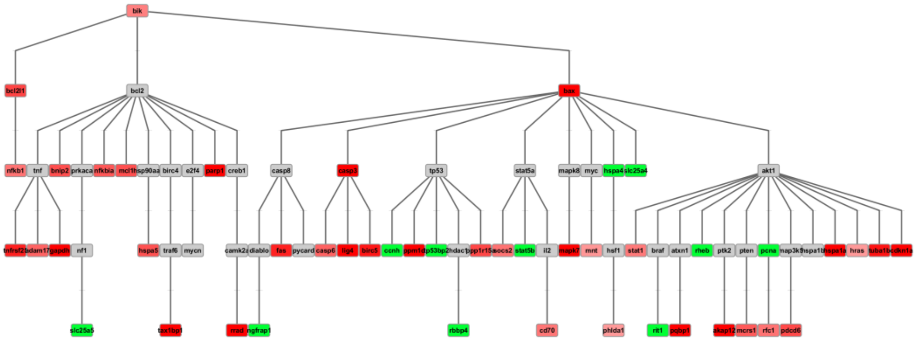

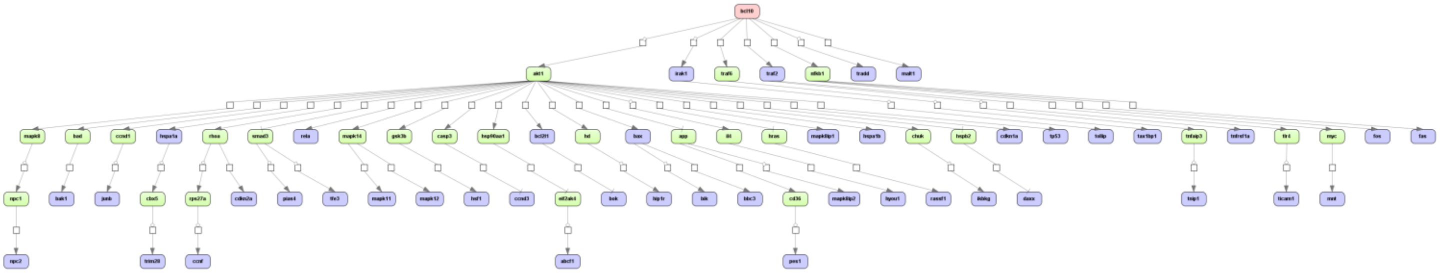

96h BIK with DEG

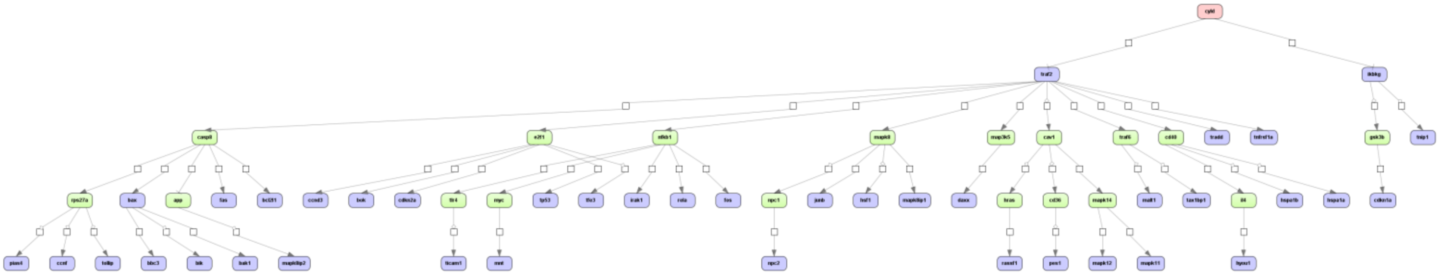

Top 3 regulators 48h bcl10

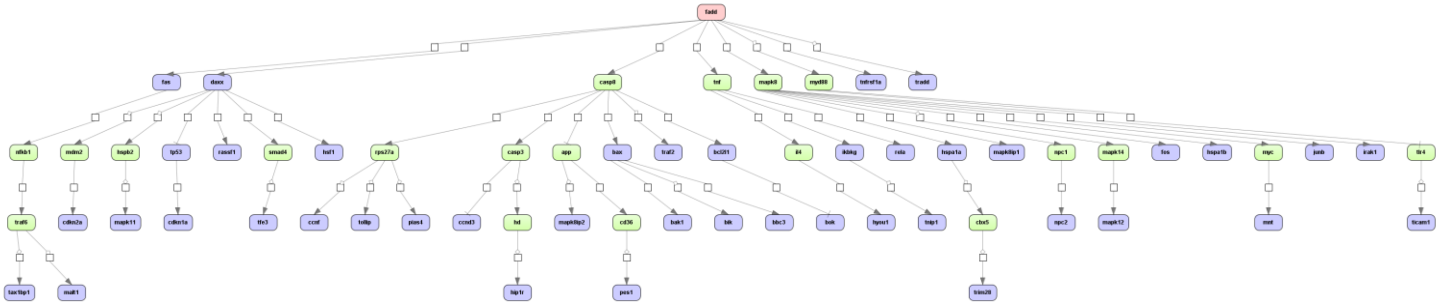

Top 3 regulators 48h cyld

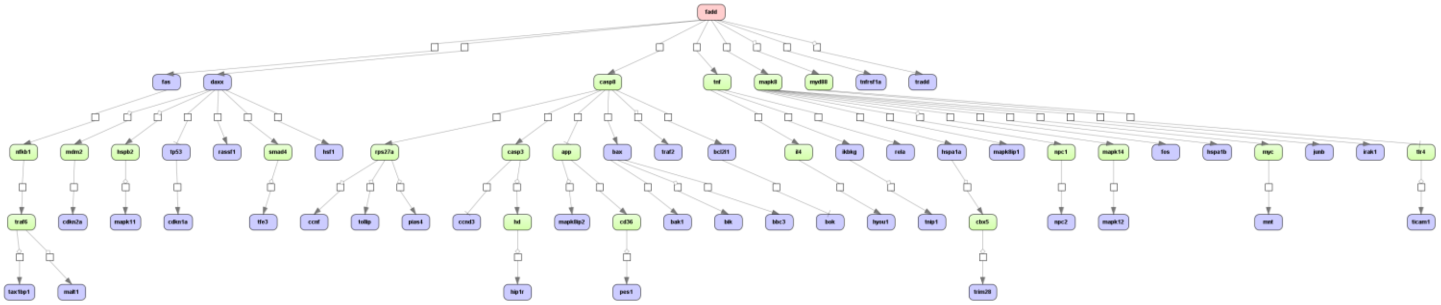

Top 3 regulators 48h fadd

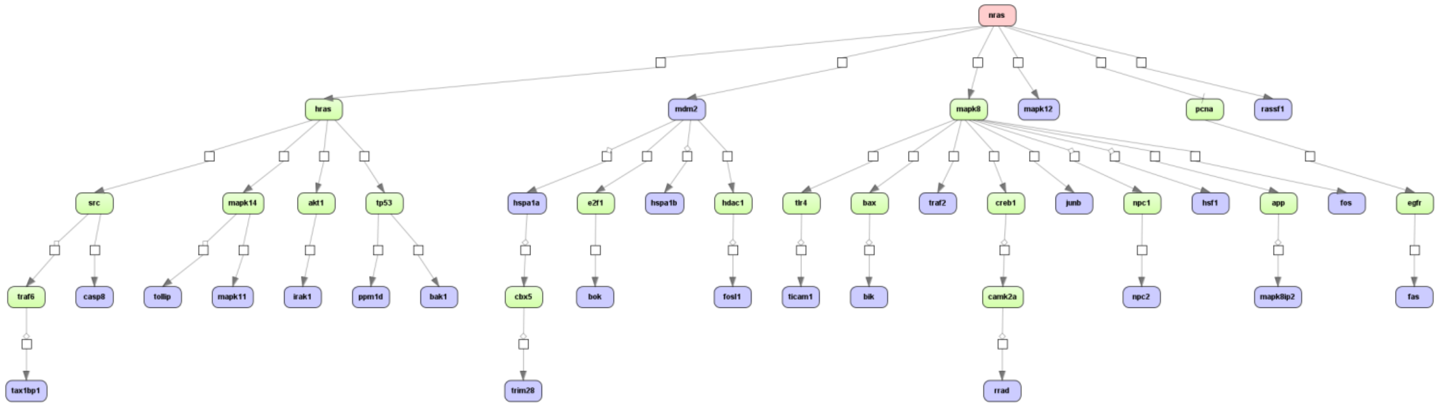

Top 3 regulators 72h nras

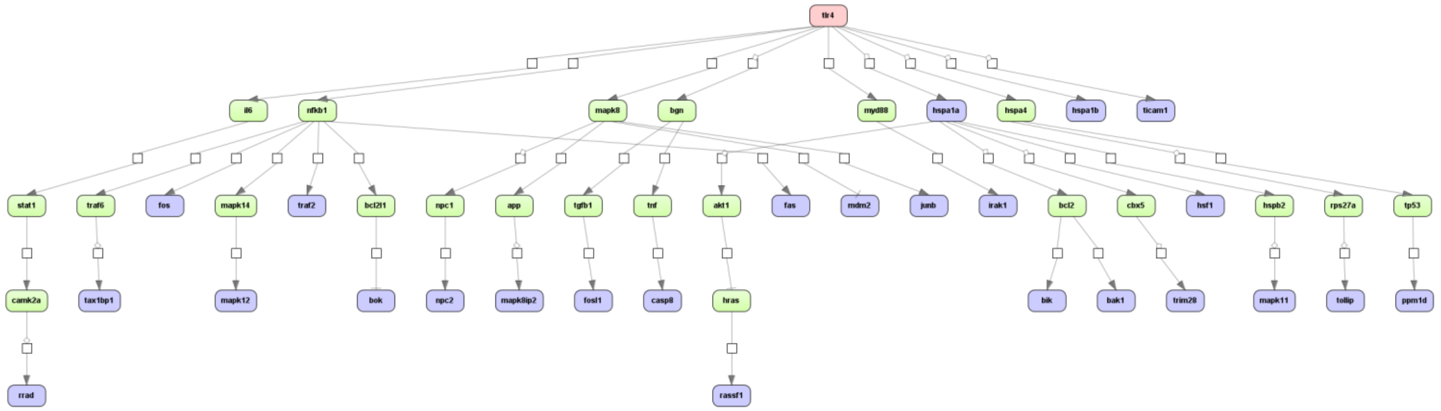

Top 3 regulators 72h tlr4

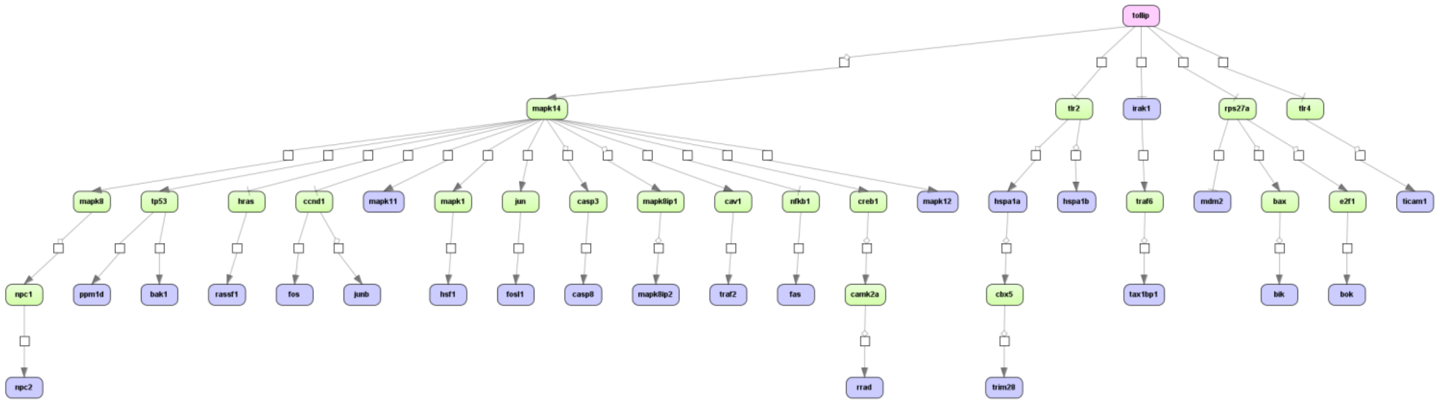

Top 3 regulators 72h tollip

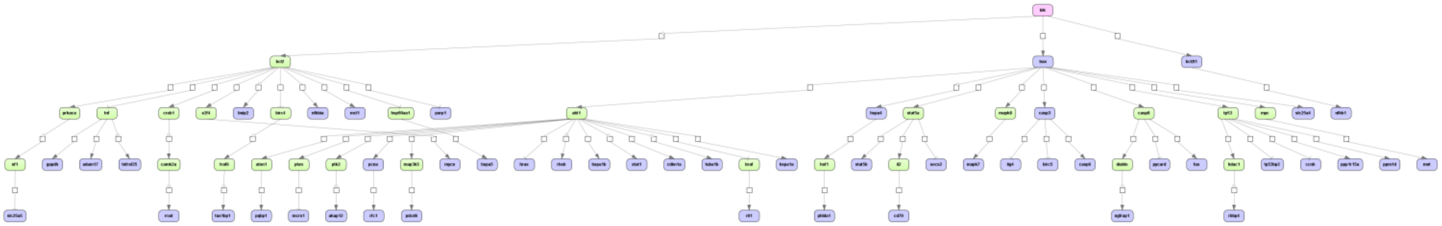

Top 3 regulators 96h bik

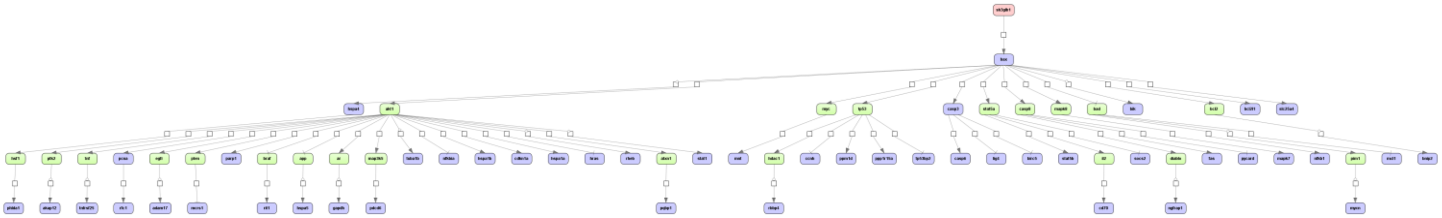

Top 3 regulators 96h sh3glb1

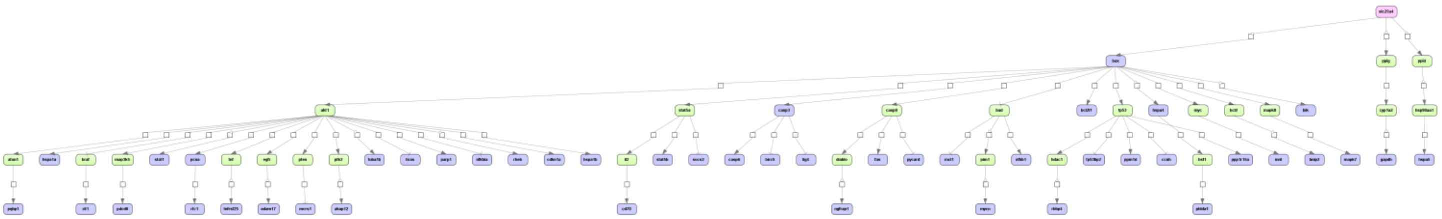

Top 3 regulators 96h slc25a4
