# Supplementary material for: Time-course gene profiling and networks in demethylated retinoblastoma cell line
Source: Oncotarget. 2015 Jun 25;6(27):23688–707. doi: 10.18632/oncotarget.4644 (PMC4695145; doi:10.18632/oncotarget.4644)
Supplement: Supplementary file 2 [file oncotarget-06-23688-s002.pdf]

Time-course gene profiling and networks in demethylated retinoblastoma cell line

Supplementary Material

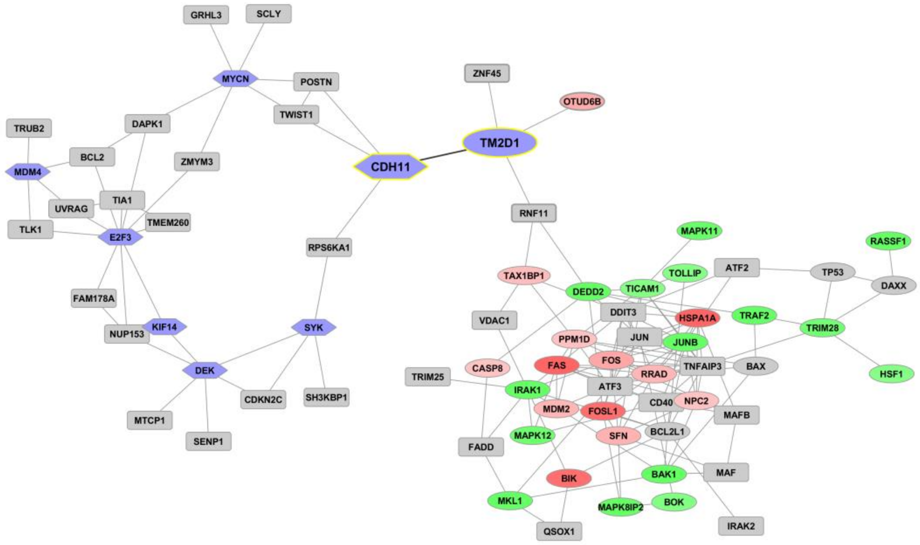

Supplementary Figure 1

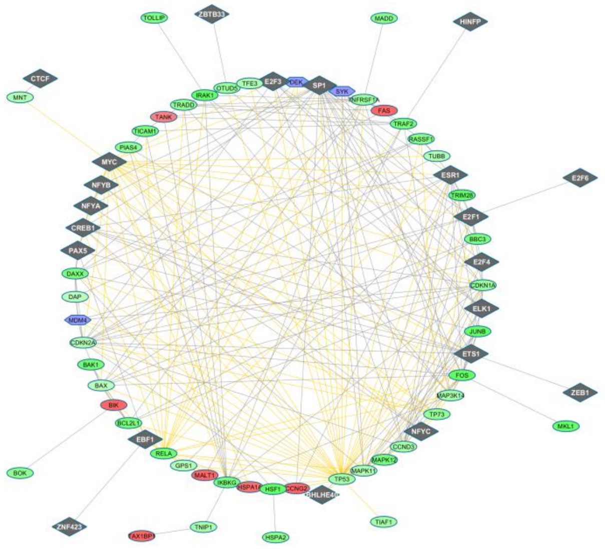

Supplementary Figure 2

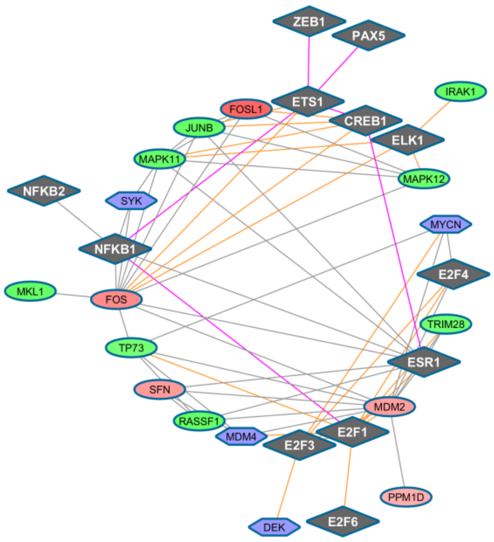

Supplementary Figure 3

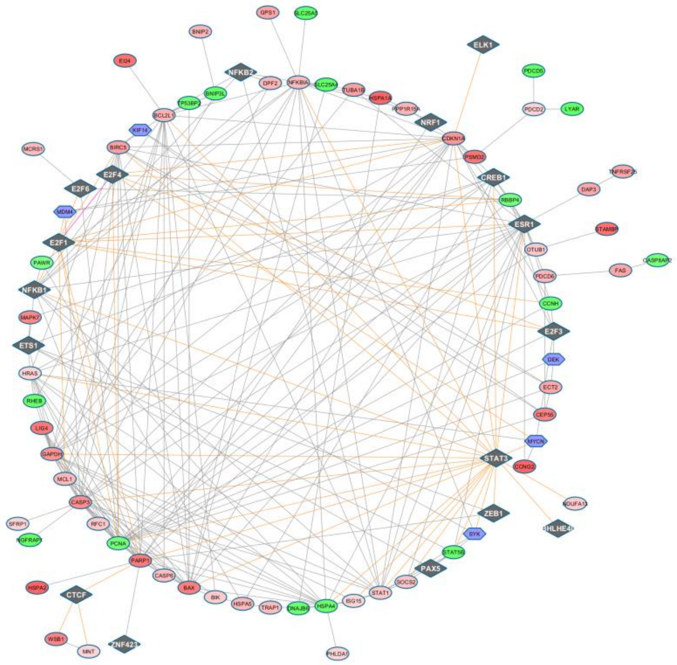

Supplementary Figure 4

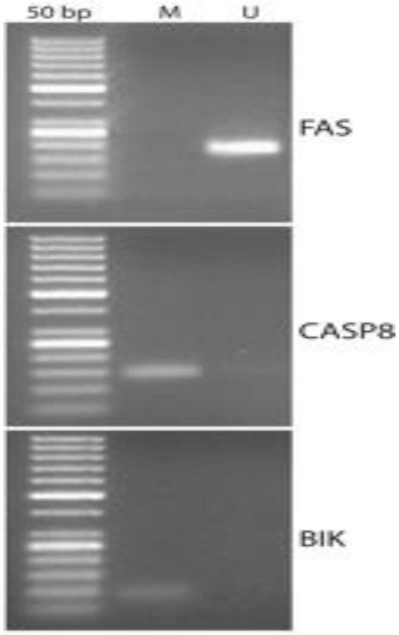

Supplementary Figure 5

MSP Wery Rb1 cells.

Ethidium bromide stained agarose gels showing PCR products obtained with primers for specific methylated (M) and unmethylated (U) promoter sequences. The methylation status of the same region of selected genes (FAS, CASP8 and BIK), involved on apoptotic signalling, is shown for Weri-Rb1 cells.
